# [Arg^6^,D-Trp^7,9^,N^me^Phe^8^]-substance P (6–11) activates JNK and induces apoptosis in small cell lung cancer cells via an oxidant-dependent mechanism

**DOI:** 10.1038/sj.bjc.6690458

**Published:** 1999-06

**Authors:** A C MacKinnon, R A Armstrong, C M Waters, J Cummings, J F Smyth, C Haslett, T Sethi

**Affiliations:** Rayne Laboratory, Respiratory Medicine Unit, University of Edinburgh Medical School, Teviot Place, Edinburgh EH8 9AG, UK; Dietetics and Nutrition, Queen Margaret College, Clerwood Terrace, Edinburgh EH12 8TS, UK; Imperial Cancer Research Fund, Medical Oncology Unit, Western General Hospital, Crewe Road South, Edinburgh EH4 2XU, UK

**Keywords:** antagonist G, SCLC, JNK, apoptosis, G-protein, neuropeptide

## Abstract

[Arg^6^,D-Trp^7,9^,N^me^Phe^8^]-substance P (6–11) (antagonist G) is a novel class of anti-cancer agent that inhibits small-cell lung cancer (SCLC) cell growth in vitro and in vivo and is entering phase II clinical investigation for the treatment of SCLC. Although antagonist G blocks SCLC cell growth (IC_50_ = 24.5 ± 1.5 and 38.5 ± 1.5 μM for the H69 and H510 cell lines respectively), its exact mechanism of action is unclear. This study shows that antagonist G stimulates apoptosis as assessed by morphology (EC_50_ = 5.9 ± 0.1 and 15.2 ± 2.7 μM for the H69 and H510 cell lines respectively) and stimulates c-*jun*-N-terminal kinase (JNK) activity in SCLC cells (EC_50_ = 3.2 ± 0.1 and 15.2 ± 2.7 μM). This activity is neuropeptide-independent, but dependent on the generation of reactive oxygen species (ROS) and is inhibited by the free radical scavenger n-acetyl cysteine. Furthermore, antagonist G itself induces inflammation (59% increase in oedema volume compared to control) and potentiates (by 35–40%) bradykinin-induced oedema formation in vivo. In view of these results we show that, as well as acting as a ‘broad-spectrum’ neuropeptide antagonist, antagonist G stimulates basal G-protein activity in SCLC cell membranes (81 ± 12% stimulation at 10 μM), thereby displaying a unique ability to stimulate certain signal transduction pathways by activating G-proteins. This novel activity may be instrumental for full anti-cancer activity in SCLC cells and may also account for antagonist G activity in non-neuropeptide-dependent cancers. © 1999 Cancer Research Campaign


British Journal of Cancer (1999) **80**(7), 1026–1034
© 1999 Cancer Research Campaign
Article no. bjoc.1998.0458

### [$Arg^6$,D-$Trp^{7,9}$,$N^{me}Phe^8$]-substance P (6–11) activates JNK and induces apoptosis in small cell lung cancer cells via an oxidant-dependent mechanism

**AC MacKinnon[1], RA Armstrong[2], CM Waters[3], J Cummings[3], JF Smyth[3], C Haslett[1] and T Sethi[1]**

[1]Rayne Laboratory, Respiratory Medicine Unit, University of Edinburgh Medical School, Teviot Place, Edinburgh EH8 9AG, UK; [2]Dietetics and Nutrition, Queen Margaret College, Clerwood Terrace, Edinburgh EH12 8TS, UK; [3]Imperial Cancer Research Fund, Medical Oncology Unit, Western General Hospital, Crewe Road South, Edinburgh EH4 2XU, UK

**Summary** [$Arg^6$,D-$Trp^{7,9}$,$N^{me}Phe^8$]-substance P (6–11) (antagonist G) is a novel class of anti-cancer agent that inhibits small-cell lung cancer (SCLC) cell growth in vitro and in vivo and is entering phase II clinical investigation for the treatment of SCLC. Although antagonist G blocks SCLC cell growth ($IC_{50}$ = 24.5 ± 1.5 and 38.5 ± 1.5 μM for the H69 and H510 cell lines respectively), its exact mechanism of action is unclear. This study shows that antagonist G stimulates apoptosis as assessed by morphology ($EC_{50}$ = 5.9 ± 0.1 and 15.2 ± 2.7 μM for the H69 and H510 cell lines respectively) and stimulates c-*jun*-N-terminal kinase (JNK) activity in SCLC cells ($EC_{50}$ = 3.2 ± 0.1 and 15.2 ± 2.7 μM). This activity is neuropeptide-independent, but dependent on the generation of reactive oxygen species (ROS) and is inhibited by the free radical scavenger n-acetyl cysteine. Furthermore, antagonist G itself induces inflammation (59% increase in oedema volume compared to control) and potentiates (by 35–40%) bradykinin-induced oedema formation in vivo. In view of these results we show that, as well as acting as a 'broad-spectrum' neuropeptide antagonist, antagonist G stimulates basal G-protein activity in SCLC cell membranes (81 ± 12% stimulation at 10 μM), thereby displaying a unique ability to stimulate certain signal transduction pathways by activating G-proteins. This novel activity may be instrumental for full anti-cancer activity in SCLC cells and may also account for antagonist G activity in non-neuropeptide-dependent cancers.

**Keywords:** antagonist G; SCLC; JNK; apoptosis; G-protein; neuropeptide

Lung cancer is the commonest fatal malignancy in the developed world. Small-cell lung cancer (SCLC), which constitutes 25% of the total, is a particularly aggressive form of lung cancer. It metastasises early, and over 90% of patients have widespread metastasis at presentation, precluding curative surgery. Once SCLC was understood to be a systemic disease, systemic chemotherapy became the treatment of choice. However, despite initial sensitivity to radio- and chemotherapy, SCLC almost invariably relapses, so that the 2-year survival remains less than 5% (Smyth et al, 1986). The incidence of lung cancer deaths is expected to rise even further in the next 20 years, with an increasing number of cases unrelated to smoking. The epidemic proportions of lung cancer are sharply contrasted by the general failure of conventional treatment. Novel forms of treatment are urgently required.

SCLC proliferation is driven by multiple autocrine and paracrine growth loops involving calcium-mobilizing neuropeptides, including bradykinin, cholecystokinin, galanin, gastrin, gastrin-releasing peptide (GRP), neurotensin and vasopressin (Moody et al, 1981; Cuttitta et al, 1985; Zachary et al, 1986; Sethi and Rozengurt, 1991). The binding of these peptides to their receptors is a critical factor in the aggressive growth of SCLC (Woll and Rozengurt, 1990; Sethi and Rozengurt, 1991*a*; Sethi et al, 1992). In SCLC cells, neuropeptides acting via $G_q$ stimulate a rise in intracellular $Ca^{2+}$ via the activation of phospholipase C (PLC) and subsequent generation of inositol phosphates (Woll and Rozengurt, 1989; Sethi and Rozengurt, 1991*a*, 1991*b*). Neuropeptides also activate the mitogen-activated protein kinase (MAPK) pathway via a protein kinase C-dependent mechanism (Seufferlein and Rozengurt, 1996; Seckl et al, 1997). These effects result in the activation of transcription factors leading to an overall stimulation of cell growth and proliferation (for review see Blumer and Johnson, 1994). Interruption of the mitogenic second messenger signals initiated by these neuropeptides could offer a novel and effective form of treatment to prevent SCLC cell growth.

Studies on the pharmacology of a family of substance P analogues have shown that these compounds inhibit the effects of a broad range of neuropeptides that are structurally unrelated to substance P and hence have been termed 'broad-spectrum' neuropeptide anatgonists (Woll and Rozengurt, 1990; Sethi et al, 1992). These compounds inhibit SCLC cell growth in vivo and in vitro (Woll and Rozengurt, 1988; Sethi et al, 1992; Seckl et al, 1997). In particular, [$Arg^6$,D-$Trp^{7,9}$,$N^{me}Phe^8$]-substance P (6–11) (antagonist G) is now entering phase II clinical investigation for the treatment of SCLC. Understanding the precise mechanism of action of antagonist G is important for the design of new anti-cancer agents and for future clinical use but, despite the clinical importance and the advanced stage of clinical investigation, the precise mechanism of action of these compounds remains unclear and controversial. Studies in Swiss 3T3 cells suggested that substance P analogues competitively inhibit the binding of neuropeptides to their receptors (Woll and Rozengurt, 1988; Seckl et al, 1995, 1997), accounting for the inhibition of neuropeptide-stimulated $Ca^{2+}$ mobilization (Woll and Rozengurt, 1990), MAPK activity and growth in both Swiss 3T3 fibroblasts and SCLC cells

*Received 30 September 1998*
*Revised 17 December 1998*
*Accepted 18 December 1998*

*Correspondence to:* T Sethi

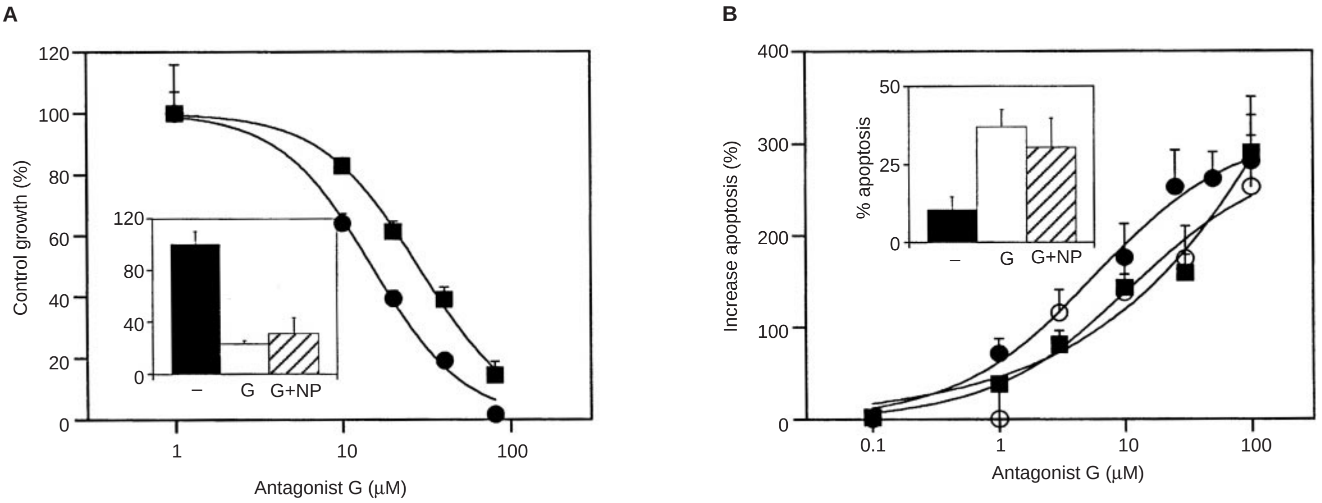

**Figure 1** Effect of antagonist G on growth and apoptosis. (**A**) *Growth*: NCI-H69 (closed circles) and NCI-H510 (closed squares) SCLC cells were quiesced overnight, washed and resuspended in SITA medium at a density of $5 \times 10^4$ cells per ml in the presence of 0–80 μM antagonist G. Total cell number was determined using a Coulter counter (Coulter Electronics). The data is expressed as % control growth in the absence of antagonist G and each point represents the mean ± s.e.m. of three experiments performed in duplicate. The cell number at day 9 was used for $IC_{50}$ determinations. The inset shows the effect of 50 μM antagonist G in H69 cells in the absence (G) and presence (G+NP) of bradykinin, neurotensin, vasopressin and cholecystokinin all at 1 μM. (**B**) *Apoptosis*: SCLC cells NCI-H69 (closed circles) and H510 (closed squares) and Swiss 3T3 cells (open circles) were washed and incubated with antagonist G for 24 h at 37°C. Apoptosis was assessed morphologically as described (Tallet et al, 1996). The data are expressed as % increase in apoptosis and represents the mean ± s.e.m. of four independent experiments performed in triplicate. The inset shows the effect of 50 μM antagonist G in H69 cells on basal apoptosis in the absence (G) and presence (G+NP) of bradykinin, neurotensin, vasopressin and cholecystokinin all at 1 μM

(Seckl et al, 1995, 1996, 1997). However, as well as having direct receptor effects, short hydrophobic peptides including substance P are able to cross or insert into cell membranes, and consequently are able to promote G-protein-mediated activation of PLC and other effectors (Mousli et al, 1990). In addition, some substance P analogues can inhibit receptor/G-protein coupling by binding directly to the G-protein (Mukai et al, 1992). Thus, the basis for the action of substance P analogues is complex.

Withdrawal of growth factors from both normal and tumour cells results in a specific, and highly organized, form of cell death termed apoptosis (Kerr et al, 1972; Kyprianou et al, 1992). Factors affecting the balance between SCLC cell proliferation and apoptosis will have a profound effect on tumour growth. However, the mechanisms regulating apoptosis remain poorly understood. The p46/p54 c-*jun* N-terminal kinases (JNKs) are members of the MAPK family which activate the transcription factors c-*jun* and ATF2, and are stimulated by environmental stress (e.g. heat shock, ultraviolet, tumour necrosis factor (TNF)-α, receptor tyrosine kinases and G-protein-linked receptors (Coso et al, 1995*a*; Chen et al, 1996; Verheij et al, 1996). Activation of JNK1 has been shown to be important for UV-induced apoptosis in SCLC cells (Butterfield et al, 1997). Reactive oxygen species (ROS) have been shown to be involved in events leading to apoptosis in many cell types (Buttke and Sandstrom, 1994). ROS have been shown to activate JNK (Laderoute and Webster, 1997), c-*jun* expression and AP-1 activity (Janssen et al, 1997; Xu et al, 1997). The role of ROS in pathways leading to programmed cell death is particularly pertinent in cancers where the oxygen tension at the centre of tumours may be particularly low (Bush et al, 1978).

In this study we show that, as well as inhibiting neuropeptide responses, antagonist G has neuropeptide-independent agonist activity in SCLC cells. The agonist activity is mediated via free radical oxygen formation leading to JNK activation, which may be important for antagonist G-induced apoptosis of SCLC cells in vitro. We suggest that both of these effects are likely to be crucial for antagonist G's maximal anti-tumour effect in vivo.

### MATERIALS AND METHODS

### Materials

Swiss 3T3 fibroblasts and SCLC cell lines NCI-H69 and NCI-H510 were purchased from the American Type Tissue Culture Collection (Rockville, MD, USA); RPMI-1640 and DMEM were obtained from Sigma (Poole, UK); antagonist G was a kind gift from Peptec (Copenhagen, Denmark); male New Zealand White rabbits were purchased from Charles River (Kent, UK); [$^{35}$S]-GTPγS (1000 Ci mmol$^{-1}$), [γ$^{32}$P]-ATP (3000 Ci mmol$^{-1}$) and [$^{125}$I]-albumin (2.5 μCi mg$^{-1}$) were purchased from Amersham plc (Amersham, UK); JNK1-FL, p42$^{MAPK}$ polyclonal antibodies, GST c-*jun*(79), myelin basic protein and protein A/G agarose were purchased from Santa Cruz Biotechnology (Santa Cruz, CA, USA). All other reagents were of the purest grade available.

### Cell culture

NCI-H69 and NCI-H510 SCLC cells were cultured in RPMI-1640 medium with 25 mM HEPES supplemented with 10% (v/v) fetal calf serum, 50 U ml$^{-1}$ penicillin, 50 μg ml$^{-1}$ streptomycin and 5 μg ml$^{-1}$ L-glutamine. Cell cultures were allowed to grow to a density of $10^6$ per ml in a humidified atmosphere of 5% carbon dioxide 95% air at 37°C. For experimental purposes, the cells were grown in SITA medium consisting of RPMI-1640 medium supplemented with 30 nM selenium, 5 μg ml$^{-1}$ insulin, 10 μg ml$^{-1}$ transferrin and 0.25% (w/v) bovine serum albumin (BSA). Cells were quiesced in serum-free RPMI-1640 medium containing 0.25% (w/v) BSA. In anoxic studies, cells were incubated at 37°C in a MK3 anaerobic incubator with 0% oxygen (Don Whitely Scientific Ltd, Yorkshire, UK).

© 1999 Cancer Research Campaign 

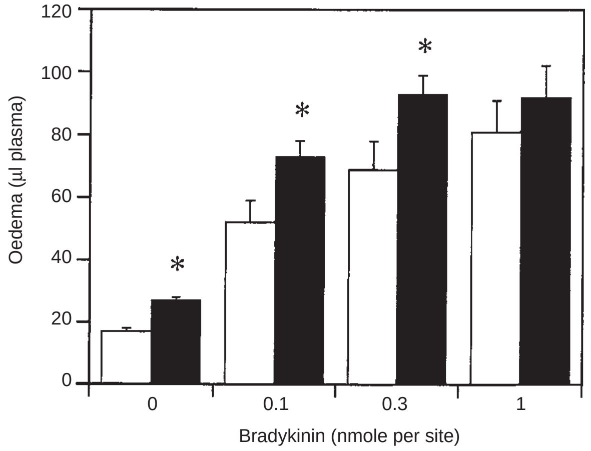

**Figure 2** Effect of antagonist G on oedema formation in the rabbit skin. Bradykinin (0.1–1.0 nmole per site) was injected in the absence (open bars) or presence (closed bars) of antagonist G (50 nmole per site). Oedema formation after 30 min was measured as μl plasma exudate as described in Methods. The results represent the mean ± s.e.m. of 4–8 experiments. *Statistically different from control $P < 0.001$ ANOVA

Swiss 3T3 cells were maintained in Dulbecco's modified Eagles medium (DMEM) containing 10% (v/v) fetal calf serum, 50 U $ml^{-1}$ penicillin, 50 μg $ml^{-1}$ streptomycin and 5 μg $ml^{-1}$ L-glutamine.

### SCLC growth

SCLC cells, 3–5 days post-passage, were washed and resuspended in SITA medium at a density of $5 \times 10^4$ cells per ml in the presence of 0–80 μM antagonist G. At each time point cell clusters were disaggregated by passing the cell suspension through 19- and 21-gauge needles and the total cell number was determined using a Coulter counter (Coulter Electronics).

### Measurement of apoptosis

SCLC cells were cultured in SITA medium and quiesced overnight in serum-free medium. SCLC cells ($10^6$ cells per ml) or quiescent cultures of Swiss 3T3 cells in 33-mm plates were incubated in the presence or absence of antagonist G or other test agents for 24 h. Adherent and non-adherent cells were cytocentrifuged onto glass slides, fixed with methanol, stained with May–Grünwald–Giemsa stain and examined using an Olympus BH-2 microscope, at a magnification of ×400. Apoptotic SCLC cells displayed typical morphological features, including cell shrinkage and chromatin condensation as previously detailed (Tallet et al, 1996). The percentage of apoptotic cells was estimated from > 500 cells counted from 4–5 random fields.

### Rabbit model of inflammation

Oedema formation was measured using a rabbit skin model of inflammation (Williams, 1979; Armstrong et al, 1995). Briefly, New Zealand white rabbits (male, 2–3 kg) were anaesthetized with sagatal (30 mg $kg^{-1}$ intravenously (i.v.)). The backs were shaved and marked out with four latin squares of 15 injection sites. [$^{125}$I]-Albumin (5 μCi Amersham) made up in Evans blue dye solution (2.5% (v/v) in sterile saline) was injected i.v. 5 min before inflammatory mediators were injected intradermally (i.d.) into the rabbit skin. After 30 min, the animal was sacrificed with an overdose of anaesthetic, and a blood sample (10 ml) taken by cardiac puncture. This was centrifuged (450 ***g***, 30 min) to give three aliquots (1 ml) of

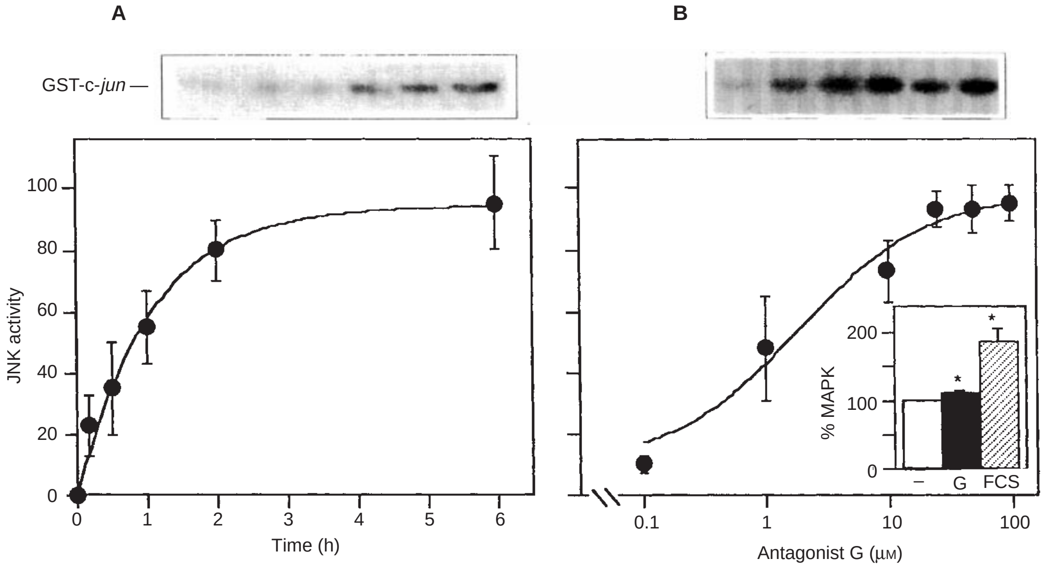

**Figure 3** Effect of antagonist G on MAPK and JNK activity in SCLC cells. (**A**) *Time course of JNK activation.* H69 cells ($1 \times 10^7$ cells per ml) were incubated with 25 μM antagonist G at 37°C for the times indicated. JNK activity was assessed by immunoprecipitation of the cell lysates with a polyclonal anti-JNK1 antibody and phosphorylation of GST–c-*jun* as described in Materials and Methods. The data are expressed as % maximal response and represent the mean ± s.e.m. of three experiments performed in duplicate. The basal JNK activity did not change significantly during the incubation (data not shown). A representative autoradiograph is shown. (**B**) *Concentration dependence of JNK activation.* H69 cells were incubated with antagonist G at the concentrations indicated for 60 min at 37°C. The results are expressed as % maximal response and represent the mean ± s.e.m. of 2–4 experiments performed in duplicate. A representative autoradiograph is shown. **Inset** *MAPK activity.* H69 cells were incubated with 50 μM antagonist G (G) for 20 min or 10% (v/v) fetal calf serum (FCS) for 10 min at 37°C. $p42^{MAPK}$ was immunoprecipitated from cell lysates and activity was measured as described in Materials and Methods. The data are expressed as % control and represent the mean ± s.e.m. of three experiments performed in duplicate

© 1999 Cancer Research Campaign plasma. Injection sites (16-mm) were punched out, counted in a γ-counter, and oedema expressed as μl equivalents of plasma exudate.

### Assay of MAPK and JNK activity

SCLC cells were cultured in SITA medium ($5 \times 10^6$ per ml), washed twice in phosphate-buttered saline (PBS) (pH 7.4), and incubated for various times with test agents as indicated in the Figure legends. MAPK activity was determined as described previously (Seckl et al, 1996; Seufferlein and Rozengurt, 1996), by immunoprecipitation with a polyclonal $p42^{MAPK}$ antibody and myelin basic protein as substrate. Assessment of immunocomplexed JNK activity was carried out essentially as described (Coso et al, 1995*a*) with minor modifications. Briefly, JNK1 was immunoprecipitated from cell lysates using 3 μg of a specific polyclonal antibody to JNK1 (Santa Cruz C-15 FL). Following precipitation with protein A/G agarose, the beads were washed twice, suspended in 20-μl kinase buffer (20 mM HEPES, pH 7.5, 7.5 mM magnesium chloride, 0.5 mM EGTA, 12.5 mM β-glycerophosphate, 0.5 mM sodium fluoride, 0.5 mM sodium orthovanadate and 2 mM dithiothreitol) and incubated at 30°C for 20 min with 20 μM ATP, 1 μCi [$\gamma^{32}$P]-ATP (3000 Ci $mmol^{-1}$) and 1 μg GST-c-*jun*(79) substrate. Phosphorylated c-*jun* was identified from autoradiographs of Coomasie blue stained sodium dodecyl sulphate polyacrylamide gel electrophoresis (SDS-PAGE) gels, and quantified by optical densitometry.

### Membrane preparation

SCLC cells cultured in SITA medium, were washed twice in ice-cold PBS. The cells were lysed in ice-cold lysis buffer (10 mM Tris–HCl pH 7.4, 5 mM EDTA, 5 mM EGTA, 1 mM phenylmethyl sulphoxide) and briefly homogenized using a Polytron tissue homogenizer (2 × 5 s setting 3). After centrifugation at 500 ***g*** for 4 min the supernatant was centrifuged at 49 000 ***g*** for 15 min at 4°C and the pellet washed twice by repeated homogenization and centrifugation in lysis buffer. The final pellet was suspended in 20 mM HEPES (pH 7.4), adjusted to 1 mg $ml^{-1}$ protein, and stored at –80°C. Protein was determined using Pierce BCA protein assay reagent (Pierce, UK).

### [$^{35}$S]-GTPγS binding

[$^{35}$S]-Guanosine 5′-*O*-(γ-thio)triphosphate ([$^{35}$S]-GTPγS) binding was carried out essentially as previously described (Weiland and Jakobs, 1994). Briefly, SCLC cell membranes (~10 μg protein) were incubated in a final volume of 100 μl binding buffer (20 mM HEPES, pH 7.4, 100 mM sodium chloride, 3 mM magnesium chloride, 10 μM guanosine 5′-diphosphate (GDP), 0.2 mM ascorbate) with 0.2 nM [$^{35}$S]-GTPγS for 60 min at 4°C in the presence or absence of test compounds. Bound radioactivity was determined by filtration onto GF-B glass fibre filters using a Brandel Cell Harvester and counted by scintillation spectrometry. Non-specific binding was determined in the presence of 100 μM unlabelled GTPγS.

### Statistical analysis

Data are presented as the mean ± s.e.m. of at least three separate determinations. Statistical significance from corresponding time control was estimated by a two-way ANOVA with significance being taken at $P < 0.05$.

### RESULTS

### Antagonist G-induced stimulation of apoptosis and inhibition of growth in SCLC cells is not reversed by neuropeptides

Antagonist G profoundly inhibited the growth of SCLC cell lines H69 and H510 in a concentration-dependent manner with $IC_{50}$ values of 24.5 ± 1.5 and 38.5 ± 1.5 μM respectively (Figure 1A). This could not be reversed by the addition of supramaximal (1 μM) concentrations of a mixture of mitogenic neuropeptides bradykinin, neurotensin, vasopressin and cholecystokinin (Figure 1A, inset). In addition, antagonist G caused a marked stimulation of apoptosis as judged morphologically. The addition of antagonist G (25 μM for 24 h) to SCLC cells cultured in SITA medium ($1 \times 10^5$ cells per ml) or Swiss 3T3 cells in 33-mm dishes caused a marked stimulation of apoptosis (Figure 1B). In the SCLC cell line H69, antagonist G increased basal apoptosis from 10.5 ± 4.2% to 37.1 ± 5.4% ($n = 4$) with an $EC_{50}$ of 5.9 ± 0.1 μM ($n = 4$; Figure 1B). Antagonist G also induced apoptosis in the SCLC cell line H510 with an $EC_{50}$ value of 15.2 ± 2.7 μM, and in Swiss 3T3 cells with an $EC_{50}$ of 11.2 ± 3.2 μM (Figure 1B). The induction of apoptosis in SCLC cells by antagonist G was confirmed using acridine orange and propidium iodide staining (not shown). As with the effects on growth, antagonist G-induced apoptosis in H69 cells could not be reversed by co-incubation with high concentrations (1 μM) of a mixture of mitogenic neuropeptides (bradykinin, neurotensin, vasopressin and cholecystokinin, Figure 1B, inset).

### Antagonist G induces oedema formation in the rabbit skin

Although antagonist G has been proposed to inhibit SCLC cell growth by inhibiting neuropeptide growth factor responses, it has never been shown to act as a neuropeptide antagonist in vivo. To examine the effects of antagonist G in vivo, a rabbit skin model of inflammation was used. In this model, bradykinin induces inflammation by acting directly on endothelial cells to cause an increase in microvascular permeability (Armstrong et al, 1995). However, rather than inhibiting bradykinin, antagonist G (50 nmol per site) significantly potentiated (ANOVA, $P < 0.001$, $n = 4$) oedema formation induced by bradykinin (Figure 2), giving a 40% potentiation at 0.1 nmole per site bradykinin. Furthermore, antagonist G itself induced oedema formation in the rabbit skin when compared to the vehicle control (27 ± 1 and 17 ± 1 μl of plasma respectively, $n = 8$, $P < 0.001$, Figure 2). Similar results were observed if antagonist G was co-injected or injected 5 min prior to the addition of bradykinin. However, unlike plasma exudation induced by bradykinin (Armstrong et al, 1995), oedema induced by this dose of antagonist G was not potentiated by the vasodilator action of prostaglandin $E_2$ ($PGE_2$) ((1 μg per site) 26 ± 1 and 27 ± 2 μl plasma in the absence and presence of of $PGE_2$, $n = 4$). Thus antagonist G does not act as a competitive broad-spectrum neuropeptide antagonist in this in vivo system.

### Antagonist G stimulates JNK activity

JNK has been shown to be an important enzyme in the transduction of apoptotic and inflammatory signals in many cell types (Coso et al, 1995*a*; Chen et al, 1996; Verheij et al, 1996). The effect of antagonist G on JNK activation was therefore examined. Antagonist G (25 μM) in the absence of neuropeptide, caused a

*© 1999 Cancer Research Campaign*

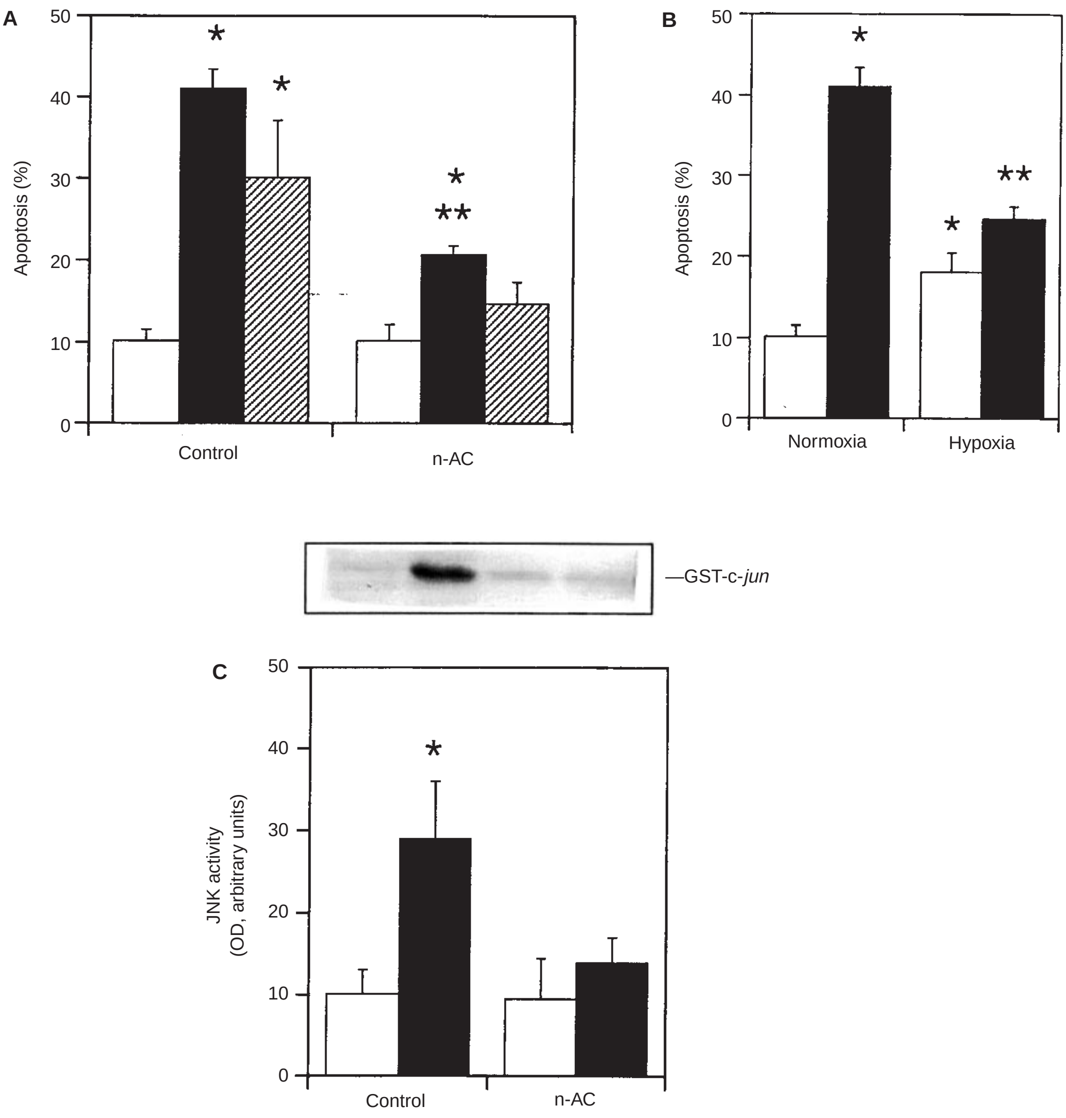

**Figure 4** Antagonist G-induced apoptosis and JNK stimulation is inhibited by anoxia and n-AC. (**A**) *Apoptosis*: H69 cells cultured in SITA medium were quiesced overnight in serum-free medium, washed and incubated ($1 \times 10^6$ cells per ml) in the absence (open bars) or presence of 25 μM antagonist G (closed bars) or 200 μM hydrogen peroxide (hatched bars) with or without 10 mM n-AC for 24 h at 37°C. Apoptosis was assessed morphologically as described in Materials and Methods. The data represent the mean ± s.e.m. of 3–4 separate experiments performed in duplicate. (**B**) Quiescent H69 cells ($1 \times 10^6$ cells per ml) were incubated under normoxic (19% $O_2$) or anoxic (MK3 anaerobic incubator, 0% $O_2$) conditions at 37°C for 24 h in the presence (closed bars) or absence (open bars) of 25 μM antagonist G. The data represents the mean ± s.e.m. of 3–4 separate experiments performed in duplicate. (**C**) *JNK activity*: Quiescent cultures of H69 cells were incubated ($1 \times 10^7$ cells per ml) in the presence (closed bars) or absence (open bars) of 25 μM antagonist G with or without 10 mM n-AC for 1 h at 37°C. JNK activity was assessed by immunoprecipitation of the cell lysates with a polyclonal anti-JNK1 antibody and phosphorylation of GST-c-*jun* as described in Materials and Methods. The data represent the mean and standard deviation of two experiments performed in duplicate. A representative autoradiogram of JNK activity is shown. *Statistically different from corresponding control $P < 0.05$; **statistically different from antagonist G control $P < 0.05$

marked and sustained activation of JNK activity in H69 cells. This effect was maximal at 2 h, and sustained for at least 6 h (Figure 3A). The activation of JNK by antagonist G was concentration-dependent (Figure 3B), with an $EC_{50}$ value of 3.2 ± 0.1 μM ($n$ = 3). Antagonist G also activated JNK in the H510 SCLC cell line in a similar manner ($EC_{50}$ = 15.2 ± 2.7 μM, data not shown). Activation of JNK by antagonist G did not occur as a result of non-specific MAPK activation, as under the same conditions that antagonist G stimulated a sustained activation of JNK, it caused only a modest increase in MAPK activity in H69 cells, which was not evident at concentrations lower than 25 μM (Figure 3B, inset). Therefore, antagonist G caused a selective stimulation of JNK over MAPK activity which may be instrumental in its ability to induce apoptosis in SCLC cells and cause inflammation.

*© 1999 Cancer Research Campaign*

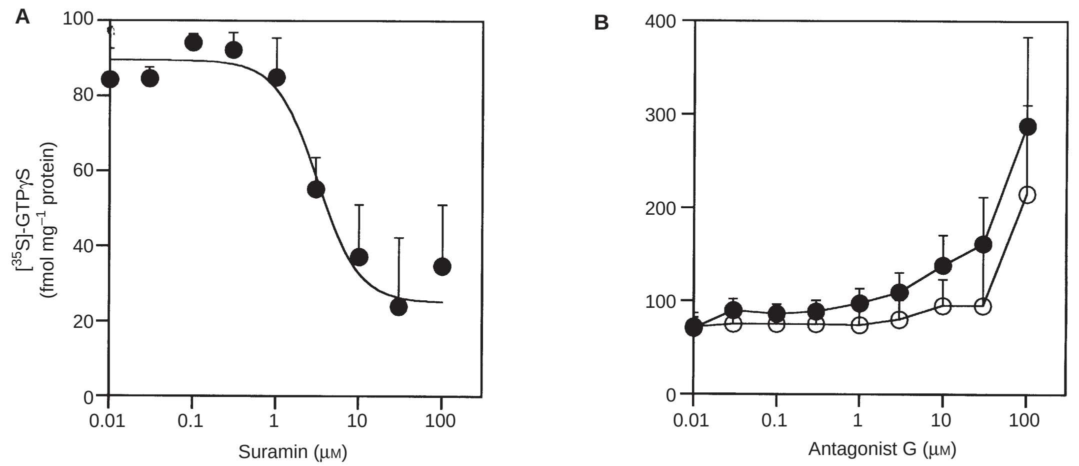

**Figure 5** Antagonist G modulates $[^{35}S]$-GTPγS binding to SCLC cell membranes. (**A**) H69 SCLC cell membranes (10 μg protein) were incubated with suramin (0.01–100 μM) and 0.2 nM $[^{35}S]$-GTPγS in the presence of 10 μM GDP and 100 mM sodium chloride for 60 min at 4°C. Bound $[^{35}S]$-GTPγS was recovered as described in Materials and Methods. The data are expressed as fmoles $[^{35}S]$-GTPγS specifically bound/mg membrane protein and represent the mean ± s.e.m. of four separate experiments performed in duplicate. (**B**) H69 SCLC cell membranes were incubated with antagonist G (0.01–300 μM) and 0.2 nM $[^{35}S]$-GTPγS in the presence (open circles) or absence (closed circles) of 1 μM d($CH_2$)-5-TyrMe-arginine vasopressin. The data represent the mean ± s.e.m. of four separate experiments performed in duplicate

### Role of ROS in apoptosis and JNK activation induced by antagonist G

The generation of free ROS is implicated in the activation of JNK and apoptosis in certain cell systems. Understanding the role of oxygen radicals is important in any new anti-cancer therapeutic agent as the centre of many tumours are often profoundly hypoxic. The role of oxidant stress on basal and antagonist G-induced SCLC cell apoptosis was therefore examined.

Addition of the oxygen donor, hydrogen peroxide (200 μM for 24 h) caused a marked stimulation in the rate of SCLC cell apoptosis which was comparable to the extent of apoptosis induced by antagonist G and was inhibited by co-incubation with the oxygen radical scavenger n-acetylcysteine (n-AC, Figure 4A). Thus, free radical oxygen can induce apoptosis in SCLC cells and this can be blocked by n-AC. Incubation of SCLC cells under anoxic conditions markedly attenuated the apoptotic effect of 25 μM antagonist G (41.1 ± 2.4% to 24.7 ± 1.5%; $n$ = 4) with only a modest rise in the background rate of apoptosis (10.2 ± 1.4% to 18.1 ± 2.4%; $n$ = 4) (Figure 4B). This suggests that antagonist G requires the generation of free ROS to induce apoptosis. This was confirmed using the free radical scavenger n-AC (Figure 4A). Furthermore, n-AC (10 mM) also markedly inhibited the antagonist G-induced activation of JNK (Figure 4C). Antagonist G did not act as an oxygen donor itself as it had no effect on cytochrome c activity, but generated oxygen radicals within the cell as measured by an increase in dihydrorhodamine fluorescence (results not shown).

### Antagonist G stimulates G-protein activity

Our results suggest that antagonist G has direct agonist activity in SCLC cells. We therefore postulated that antagonist G may stimulate G-protein activity. G-protein activity was measured in SCLC H69 cell membranes which had been well washed to ensure removal of endogenous neuropeptide. $[^{35}S]$-GTPγS binding was conducted in the presence of 10 μM GDP and 100 mM NaCl to maintain G-protein in a GDP-bound form which is required for optimal agonist activation (Weiland and Jakobs, 1994). The effect of antagonist G was compared with the anti-cancer drug suramin, which has been shown to block the activation of specific G-protein α-subunits (Freissmuth et al, 1996). Suramin produced a concentration-dependent inhibition of basal $[^{35}S]$-GTPγS binding to H69 SCLC cell membranes ($IC_{50}$ 6.8 ± 1.2 μM, $n$ = 3; Figure 5A). However, antagonist G, over the concentration range that inhibits the growth of SCLC cells in vitro and in vivo produced a dose-dependent increase in $[^{35}S]$-GTPγS binding, giving a 4.1-fold stimulation at 100 μM (Figure 5B). Antagonist G has been shown to be most potent at $V_{1A}$-vasopressin receptors (Seckl et al, 1995), it was postulated therefore that the ability of antagonist G to stimulate $[^{35}S]$-GTPγS binding to SCLC cells may be via partial agonism at $V_{1A}$ receptors. However, addition of a maximal concentration of the selective $V_{1A}$-receptor antagonist d($CH_2$)-5-TyrMe-argVP had no effect on $[^{35}S]$-GTPγS binding alone, and did not inhibit the antagonist G-induced G-protein activation (Figure 5B), suggesting that the agonist effect of antagonist G on G-protein activation is not mediated via the $V_{1A}$-receptor.

### DISCUSSION

Antagonist G is a novel and exciting therapeutic agent for SCLC. Antagonist G inhibits SCLC growth in vitro and in vivo and is currently about to enter phase II clinical investigation for the treatment of SCLC. However, despite this advanced stage of clinical investigation the exact mechanism of action is controversial. It has been proposed that unrestrained proliferation of SCLC is driven by multiple autocrine and paracrine loops involving $Ca^{2+}$-mobilizing neuropeptides (Cuttitta et al, 1985; Sethi and Rozengurt, 1991; Sethi, 1992). Neuropeptides have been shown to activate MAPK in SCLC cells (Seufferlein and Rozengurt, 1996; Seckl et al, 1997), and since antagonist G analogues inhibit neuropeptide-stimulated MAPK activity (Mitchell et al, 1995; Seckl et al, 1997), this has been suggested as a key mechanism for its anti-proliferative activity. However, we have shown that as well as inhibiting growth, antagonist G also induces apoptosis, and that both of these effects

© 1999 Cancer Research Campaign are irreversible with neuropeptides. The H69 cell line has been shown to respond to only six out of 32 neuropeptides tested (Woll and Rozengurt, 1989), and was shown to be most responsive to bradykinin and neurotensin. In our study we used supramaximal concentrations of four of the six active neuropeptides, including bradykinin and neurotensin, which should be able to reverse the effects of a submaximal concentration of antagonist G if it were acting as a competitive neuropeptide antagonist at these receptors. Previous studies have also shown that the effects of antagonist G on growth of H69 cells could not be reversed by GRP and galanin (Woll and Rozengurt, 1990) the other two neuropeptides shown to be mitogenic in these cells (Woll and Rozengurt, 1989). The inability of these mitogens to reverse the effects of antagonist G suggests a neuropeptide-independent mechanism of action. We cannot rule out the possibility that there may be another, as yet unidentified, neuropeptide receptor that is blocked by antagonist G, but we believe that to be unlikely in the light of the known repertoire of receptors in SCLC cells. So, despite the well-documented antagonistic effects of antagonist G on neuropeptide-mediated signal transduction in vitro, these may not be the only mechanisms responsible for its full anti-tumour activity.

It has been hypothesized that cells are intrinsically programmed to undergo apoptosis unless they are continuously stimulated by survival factors released from neighbouring cells (Raff et al, 1992). This is of the utmost importance in the survival and and metastasis of cancer cells, as these cells are able to establish themselves at metastatic sites in distinct locations despite the dysregulation of survival factors. Studies from this laboratory have shown this to be particularly important for the survival of SCLC cells in vivo in that extracellular matrix proteins, such as laminin and fibronectin found surrounding SCLC metastasis, are able to protect the cells from chemotherapy-induced apoptosis (Sethi et al, 1998). Therefore the stimulation of apoptosis as well as inhibition of SCLC growth would be essential for an inhibition of proliferation of SCLC in vivo.

Neuropeptide antagonism has never been described for antagonist G in vivo. To examine this, we chose a rabbit skin model of inflammation. In this system, bradykinin stimulates oedema formation via the $B_2$ bradykinin receptor (Williams, 1979; Armstrong et al, 1995), which is the same subtype involved in bradykinin-induced $Ca^{2+}$ mobilization and mitogenesis (Woll and Rozengurt, 1989; Sethi and Rozengurt, 1991*a*). However, even though antagonist G can inhibit functional responses to bradykinin in vitro, rather than inhibiting oedema, antagonist G directly induced oedema formation on its own and potentiated oedema induced by bradykinin. Moreover, the effects of antagonist G on bradykinin-induced oedema formation were additive suggesting an independent mechanism of action. This action was not related to a substance P-like effect, as substance P does not directly induce oedema in rabbit skin (Brain and Williams, 1985). Since antagonist G-induced oedema was not potentiated by the vasodilator action of $PGE_2$, our results suggest that vasodilatation itself would not account for the oedema observed with antagonist G. Thus, in this in vivo model, antagonist G was not behaving as a competitive neuropeptide receptor antagonist, but rather as an inducer of oedema formation.

We have shown that antagonist G has little effect on basal MAPK activity, while producing a sustained and concentration-dependent increase in JNK activity. Increased JNK activity is often associated with cellular stresses, such as UV exposure and heat shock, but also with receptor stimulation via proinflammatory cytokines and growth factors acting at tyrosine kinase and G-protein-linked receptors (Coso et al, 1995*a*; Chen et al, 1996; Verheij et al, 1996; Butterfield et al, 1997). Although JNK activation by cellular stress results in cell damage and apoptosis, stimulation of JNK by G-protein-linked receptors often results in cellular transformation (Prasad, 1995; Heasley et al, 1996). The finding that antagonist G stimulates JNK activity, despite blocking other signals via G-protein-linked receptors, provides further evidence that the sustained activation of JNK occurs by an alternative mechanism other than neuropeptide receptor antagonism. It has been suggested that the coordinated activation of the MAPK and the JNK pathway is important for cell survival, whilst the sustained activation of JNK in isolation results in cell death (Xia et al, 1995; Chen et al, 1996). Results from this study would lend support for this hypothesis, with antagonist G shifting the balance towards JNK activation and apoptosis. Activation of JNK may therefore play an important role in inducing apoptosis in SCLC cells in vivo and may also be important for the proinflammatory effects of antagonist G seen in rabbit skin.

We show that the activation of JNK and apoptosis by antagonist G is inhibited by antioxidants and suggest that ROS are involved in the signalling pathway leading from the receptor/G-protein to JNK. Previous studies have implicated the heterotrimeric G-proteins $G_{12}$ and $G_{13}$ as being important in the G-protein-linked receptor activation of JNK in a pathway involving the small GTPases *rac*/*cdc*42 (Coso et al, 1995*b*; Prasad et al, 1995). Moreover, in Jurkat T-cells, ROS have been shown to activate JNK in a pathway involving *ras*, *racl* or *cdc*42 as redox sensors (Lander et al, 1996), and in cardiac myocytes, hypoxia followed by reoxygenation stimulated JNK which was inhibited by n-AC (Laderoute and Webster, 1997). It has also been demonstrated that activation of $G_{12}/G_{13}$ regulates MAPK pathways in a ROS-dependent fashion, which is regulated by the epidermal growth factor receptor tyrosine kinase activity (Cunnick et al, 1998; Ghola et al, 1998). $G_{13}$ has also been shown to play an important role in apoptosis as transfection of constitutively activated $G_{13}$ results in apoptosis in two different cell lines (Althoefer et al, 1997). If JNK activation by antagonist G involves a stimulation of this pathway, then this may explain the inhibition of antagonist G-induced JNK activation and induction of apoptosis by ROS.

We have shown that antagonist G directly stimulates G-protein activity as assessed by increased [$^{35}$S]-GTPγS binding. These effects were entirely independent of neuropeptide growth factors. This property was not shared by suramin, an anti-cancer agent with a proposed mechanism involving growth factor antagonism and the selective antagonism of Gs (Freissmuth et al, 1996). These two antiproliferative agents therefore must have distinct mechanisms of action. A recent study has demonstrated that a close analogue of antagonist G, [D-Arg$^1$,D-Phe$^5$,D-Trp$^{7,9}$,Leu$^{11}$] substance P (antagonist D), increased JNK activity in Swiss 3T3 cells (Jarpe et al, 1998). The authors proposed the novel terminology 'biased agonism', used to describe ability of the compound to activate some GRP receptor/G-protein-mediated effects while blocking activation of others. Our results provide direct evidence for this hypothesis except that the antagonist G-induced JNK activation was not dependent on GRP receptors, as not only does antagonist G interact very weakly with GRP receptors (Woll and Rozengurt, 1990) but the H69 cell line does not express GRP receptors (Kado-Fong and Malfroy, 1989). Therefore, if the activation of JNK by antagonist G is receptor-dependent then it must be via a receptor/s other than the GRP receptor. Antagonist G has the highest potency

 *© 1999 Cancer Research Campaign*

for $V_{1A}$ vasopressin receptor (Seckl et al, 1995); however, we showed that the ability of antagonist G to stimulate [$^{35}$S]-GTPγS binding was not blocked by a selective $V_{1A}$ receptor antagonist suggesting that this effect was not mediated via partial agonism at the $V_{1A}$ receptor. It is well-established that, as well as having direct receptor effects, some amphiphylic peptides such as mastoparan and substance P and its analogues (e.g. antagonist E) are able to cross or insert into cell membranes and directly promote G-protein-mediated activation of PLC and other effectors (Mousli et al, 1990). Therefore, as well as interacting with neuropeptide receptors, substance P analogues are also able to act directly to modulate G-protein function. It is conceivable that, owing to its high lipophilicity (Seckl et al, 1995), antagonist G may have similar properties to modulate signal transduction directly at the G-protein level.

We suggest that, as well as inhibiting neuropeptide-mediated responses, antagonist G also activates some G-protein-mediated responses. We propose that, in SCLC cells, antagonist G stimulates $G_{12/13}$, leading to an activation of JNK, which may account for its ability to induce apoptosis. We believe that the activation of JNK by antagonist G may be crucial for its maximal anti-cancer activity, and may also be responsible for the cardiovascular toxicity seen in vivo. Whether this effect is mediated by another G-protein-linked receptor or directly at the G-protein remains to be elucidated. The identification of the specific G-proteins involved in antagonist G-modified signalling in SCLC would be useful for further drug development and provide additional targets for novel anti-tumour agents. These findings coupled with the finding that related substance P analogues can inhibit the growth of tumour lines, e.g. non-SCLC, which are not dependent on neuropeptide growth factors (Everard et al, 1992), will have important clinical implications both for the use of antagonist G and the future development of anti-proliferative and pro-apoptotic agents to treat this and other cancers.

### ACKNOWLEDGEMENTS

This work was supported in part by the Scottish Hospitals Endowment Research Trust (Grant No. SHERT 1441) and the Melville Trust for the Care and Cure of Cancer.

### REFERENCES

Armstrong RA, Marr C and Jones RL (1995) Characterization of the EP-receptor mediating dilatation and potentiation of inflammation in rabbit skin. *Prostaglandins* **49**: 205–224

Althoefer H, Eversole-Cire P and Simon MI (1997) Constitutively active Galphaq and Galpha13 trigger apoptosis through different pathways. *J Biol Chem* **272**: 24380–24386

Blumer KJ and Johnson GL (1994) Diversity in function and regulation of MAP kinase pathways. *Trends Biochem Sci* **19**: 236–240

Brain SD and Williams TJ (1985) Inflammatory oedema induced by synergism between calcitonin gene-related peptide (CGRP) and mediators of increased vascular permeability. *Br J Pharmacol* **86**: 855–860

Bush RS, Jenkin RDT, Allt WEC, Beale FA, Bean H, Dembo AJ and Pringle JF (1978) Definitive evidence for hypoxic cells influencing cure in cancer therapy. *Br J Cancer* **37**: 302–306

Butterfield L, Storey B, Maas L and Heasley LE (1997) c-*jun* $NH_2$-terminal kinase regulation of the apoptotic response of small cell lung cancer cells to ultraviolet radiation. *J Biol Chem* **272**: 10110–10116

Buttke TM and Sandstrom PA (1994) Oxidative stress as a mediator of apoptosis. *Immunol Today* **15**: 7–11

Chen Y, Meyer CF and Tan T (1996) Persistent activation of c-*jun* N-terminal kinase 1 (JNK1) in γ radiation-induced apoptosis. *J Biol Chem* **271**: 631–634

Coso OA, Chiáriello M, Kalinec G, Kyriakis JM, Woodgett J and Gutkind JS (1995*a*) Transforming G protein coupled receptors potently activate JNK (SAPK). *J Biol Chem* **270**: 5620–5624

Coso OA, Chiariello M, Yu JC, Teramoto H, Crespo P, Xu N, Miki T and Gitkind JS (1995*b*) The small GTP binding proteins racl and cdc42 regulate the activity of the JNK/SAPK signalling pathway. *Cell* **81**: 1137–1146

Cunnick JM, Dorsey JF, Standley T, Turkson J, Kraker AJ, Fry DW, Jove R and Wu J (1998) Role of tyrosine kinase activity of epidermal growth factor receptor in the lysophosphatidic acid-stimulated mitogen-activated protein kinase pathway. *J Biol Chem* **273**: 14468–14475

Cuttitta F, Carney J, Mulshine J, Moody TW, Fedorko J, Fischler A and Minna JD (1985) Bombesin-like peptides can function as autocrine growth factors in human small-cell lung cancer. *Nature* **316**: 823–826

Everard MJ, Macauley VM, Miller JL and Smith IE (1992) In vitro effects of substance P analogue [D-Arg$^1$, D-Phe$^5$, D-Trp$^{7,9}$, Leu$^{11}$] substance P on human tumour and normal cell growth. *Br J Cancer* **65**: 388–392

Freissmuth M, Boehm S, Beindl W, Nickel P, Ijzerman AP, Hohenegger M and Nanoff C (1996) Suramin analogues as subtype-selective G protein inhibitors. *Mol Pharmacol* **49**: 602–611

Gohla A, Harhammer R and Schulz G (1998) The G-protein G13 but not G12 mediates signaling from lysophosphatidic acid receptor via epidermal growth factor receptor to Rho. *J Biol Chem* **273**: 4653–4659

Heasley LE, Storey B, Fanger GR, Butterfield L, Zamarripa J, Blumberg D and Maue RA (1996) GTPase-deficient $G_{\alpha 16}$ and $G_{\alpha q}$ induce PC12 cell differentiation and persistant activation of c-*jun* $NH_2$-terminal kinases. *Mol Cell Biol* **16**: 648–656

Janssen YMW, Matalon S and Mossman BT (1997) Differential induction of c-*fos*, c-*jun* and apoptosis in lung epithelial cells exposed to ROS or RNS. *Am J Physiol* **273**: L789–L796

Jarpe MB, Knall C, Mitchell FM, Buhl A, Duzic E and Johnson GL (1998) [D-Arg$^1$, D-Phe$^5$, D D-Trp$^{7,9}$, Leu$^{11}$] substance P acts as a biased agonist towards neuropeptide and chemokine receptors. *J Biol Chem* **273**: 3097–3104

Kado-Fong H and Malfroy B (1989) Effects of bombesin on human small cell lung cancer cells: evidence for a subset of bombesin non-responsive cell lines. *J Cell Biochem* **40**: 431–437

Kerr JFK, Wyllie AH and Currie AH (1972) Apoptosis, a basic biological phenomenon with wider implications in tissue kinetics. *Br J Cancer* **26**: 239–245

Kyprianou N, English HF, Davidson NE and Isaacs JT (1992) Programmed cell death during regression of the MCF-7 human breast cancer following estrogen ablation. *Cancer Res* **51**: 162–166

Lander HM, Jacovina AT, Davis RJ and Tauras JM (1996) Differential activation of mitogen-activated protein kinases by nitric oxide-related species. *J Biol Chem* **271**: 19705–19709

Laderoute KR and Webster KA (1997) Hypoxia/reoxygenation stimulates *jun* kinase activity through redox signalling in cardiac myocytes. *Circ Res* **80**: 337–344

Mitchell FM, Heasley LE, Qian NX, Zamarripa J and Johnson GL (1995) Differential modulation of bombesin-stimulated phospholipase Cβ and mitogen-activated protein kinase activity by [D-Arg$^1$, D-Phe$^5$, D-Trp$^{7,9}$, Leu$^{11}$] substance P. *J Biol Chem* **270**: 8623–8628

Moody TW, Pert CB, Gazdar AF, Carney DN and Minna JD (1981) High levels of intracellular bombesin characterize human small-cell lung carcinoma. *Science* **214**: 1246–1248

Mousli M, Beub J, Bronner C, Rouot B and Landry Y (1990) G protein activation: a receptor-independent mode of action for cationic amphiphilic neuropeptides and venom peptides. *Trends Pharmacol Sci* **11**: 358–362

Mukai H, Munekata E and Higashijima T (1992) G protein antagonists: a novel hydrophobic peptide competes with receptor for G-protein binding. *J Biol Chem* **267**: 16237–16243

Prasad MV, Dermott JM, Heasley LE, Johnson GL and Dhanasekaran N (1995) Activation of Jun kinase/stress-activated protein kinase by GTPase-deficient mutants of $G_{\alpha 12}$ and $G_{\alpha 13}$. *J Biol Chem* **270**: 18655–18659

Raff MC (1992) Social controls on cell survival and cell death. *Nature* **356**: 397–400

Seckl MJ, Newman RH, Freemont PS and Rozengurt E (1995) Substance P-related antagonists inhibit vasopressin and bombesin but not 5′-3-O-(*thio*)triphosphate-stimulated inositol phosphate production in Swiss 3T3 cells. *J Cell Physiol* **163**: 87–95

Seckl MJ, Higgins T and Rozengurt E (1996) [D-Arg$^1$, D-Trp$^{5,7,9}$, Leu$^{11}$]substance P coordinately and reversibly inhibits bombesin and vasopressin-induced signal transduction pathways in Swiss 3T3 cells. *J Biol Chem* **271**: 29453–29460

Seckl MJ, Higgins T, Widmer F and Rozengurt E (1997) [D-Arg$^1$, D-Trp$^{5,7,9}$, Leu$^{11}$] substance P: a novel potent inhibitor of signal transduction and growth in vitro and in vivo in small cell lung cancer cells. *Cancer Res* **57**: 51–54

© 1999 Cancer Research Campaign 

Sethi T and Rozengurt E (1991*a*) Multiple neuropeptides stimulate clonal growth of small cell lung cancer: effects of bradykinin, vasopressin, cholecstoykinin, galanin, and neurotensin. *Cancer Res* **51**: 3621–3623

Sethi T and Rozengurt E (1991*b*) Galanin stimulates $Ca^{2+}$ mobilization, inositol phosphate accumulation and clonal growth in small cell lung cancer cells. *Cancer Res* **51**: 1674–1679

Sethi T, Langdon SP, Smyth JS and Rozengurt E (1992) Growth of small cell lung cancer cells, stimulation by multiple neuropeptides and inhibition by broad spectrum antagonists in vitro and in vivo. *Cancer Res* **52**: 2737s–2742s, 1992

Sethi T, Rintoul RC, Moore SM, Salter D, Choo C, Chilvers ER, Dransfield I, Donnelly S, Strieter RM and Haslett C (1998) Extracellular matrix proteins protect small cell lung cancer cells against apoptosis: a mechanism for small cell lung cancer cell growth and drug resistance in vitro. *Nature Medicine* (submitted)

Seufferlein T and Rozengurt E (1996) Galanin, neurotensin and phorbol esters rapidly stimulate activation of mitogen-activated protein kinase in small cell lung cancer cells. *Cancer Res* **56**: 5758–5764

Smyth JF, Fowlie SM, Gregor A, Crompton GK, Busutill A, Leonard RCF and Grant IWB (1986) The impact of chemotherapy on small cell carcinoma of the bronchus. *Quart J Med* **61**: 969–976

Tallet A, Chilvers ER, Hannah S, Dransfield I, Lawson MF, Haslett C and Sethi T (1996) Inhibition of neuropeptide-stimulated tyrosine phosphorylation and tyrosine kinase activity stimulates apoptosis in small cell lung cancer cells. *Cancer Res* **56**: 4255–4263

Verheij M, Bose R, Lin XH, Yao B, Jarvis WD, Grant S, Birrer MJ, Szabo E, Zon LI, Kyriakis JM, Haimovitz-Friedman A, Fuks Z and Kolesnick RN (1996) Requirement for ceramide-initiated SAPK/JNK signalling in stress-induced apoptosis. *Nature* **380**: 75–79

Weiland T and Jakobs KH (1994) Measurement of receptor-stimulated guanosine 5′-*O*-(*g*-Thio)triphosphate binding by G Proteins. *Methods Enzymol* **237**: 3–13

Williams TJ (1979) Prostaglandin E2, prostaglandin 12 and the vascular changes of inflammation. *Br J Pharmacol* **65**: 517–524

Xia Z, Dickens M, Raingeaud J, Davis RJ and Greenburg ME (1995) Opposing effects of ERK and JNK-p38 MAP kinases on apoptosis. *Science* **270**: 1326–1331

Xu Y, Bradham C, Brenner DA and Czaja MJ (1997) Hydrogen peroxide-induced liver cell necrosis is dependent on AP-1 activation. *Am J Physiol* **273**: G795–G803

Woll PJ and Rozengurt E (1988) [D-$Arg^{1}$, D-$Phe^{5}$, D-$Trp^{7,9}$, $Leu^{11}$]-substance P a potent bombesin antagonist in murine Swiss 3T3 cells, inhibits the growth of small cell lung cancer in vitro. *Proc Nat Acad Sci USA* **85**: 1859–1863

Woll PJ and Rozengurt E (1989) Multiple neuropeptides mobilise calcium in small cell lung cancer: effects of vasopressin, bradykinin, cholecystokinin, galanin and neurotensin. *Biochim Biophys Res Commun* **164**: 66–73

Woll PJ and Rozengurt E (1990) A neuropeptide antagonist that inhibits the growth of small cell lung cancer in vitro. *Cancer Res* **50**: 3868–3873

Zachary I, Sinnet-Smith JW and Rozengurt E (1986) Early events elicited by bombesin and structurally related peptides in quiescent Swiss 3T3 cells. I. Activation of protein kinase C and inhibition of epidermal growth factor binding. *J Cell Biol* **102**: 2211–2222

 *© 1999 Cancer Research Campaign*
